# The Distinct Impact of TAM Infiltration on the Prognosis of Patients With Cardia and Non-Cardia Gastric Cancer and Its Association With *H. pylori* Infection

**DOI:** 10.3389/fonc.2021.737061

**Published:** 2021-12-03

**Authors:** Min Liu, Li Zhang, Qiuru Zhou, Yuejie Wang, Qian Sun, Xiubao Ren

**Affiliations:** Department of Immunology, Tianjin Medical University Cancer Institute and Hospital, National Clinical Research Center for Cancer, Key Laboratory of Cancer Prevention and Therapy, Key Laboratory of Cancer Immunology and Biotherapy, Tianjin’s Clinical Research Center for Cancer, Tianjin, China

**Keywords:** tumor-associated macrophages, *Helicobacter pylori*, cardia gastric cancer, non-cardia gastric, location

## Abstract

**Background:**

In stage III gastric cancer (GC), the role of tumor-associated macrophages (TAMs) and *Helicobacter pylori* (*H. pylori*) infection impact tumor progression; however, the specific mechanisms remain controversial. We speculated whether this controversy is caused by differences in the location of TAM infiltration (in the core (CT) and invasive margin (MI) of primary tumors) and the topographical subsites of GC (cardia and non-cardia). Therefore, in this study, we investigated TAMs in different locations and *H. pylori* infection status as prognostic biomarkers for GC.

**Methods:**

Immunohistochemical staining for CD68 (pan-macrophage), CD163 (M2-like macrophage), and *H. pylori* in 200 samples (100 cases of cardia-GC [CGC] and 100 cases of non-cardia GC [NCGC]) was performed. We compared the number of CD68^+^ and CD163^+^ macrophages that infiltrated the CT and MI in patients with the prognosis of CGC and NCGC, respectively. In addition, we analyzed the relationship between *H. pylori* status and the prognosis of patients with GC in different locations, as well as the correlation with TAM infiltration.

**Results:**

The distribution of TAMs had distinct characteristics in CGC and NCGC, especially differences between CT and MI subtype. A Kaplan–Meier analysis showed that a high number of CD68^+^ macrophages that infiltrated the CT in CGC was associated with a better prognosis, whereas infiltration at the MI in NCGC indicated a poor prognosis. Furthermore, a high number of CD163^+^ macrophages infiltrating the MI resulted in a poor prognosis in CGC and NCGC cohorts. Considering the larger differences in the relationship between the infiltration of CD68^+^ macrophages at different locations and prognosis, we divided the GC cases into marginal and central GC, based on this difference. This resulted in an accurate estimation of the prognosis. Moreover, positive *H. pylori* status in central GC was significantly associated with a better prognosis and TAM infiltration.

**Conclusion:**

TAMs in different locations and *H. pylori* status were identified as independent prognostic markers, with an obvious correlation between them. Therefore, it is important to clarify the impact of TAM location on the prognosis of patients with GC, which contributes to the development of potential therapeutic strategies.

## Introduction

Gastric cancer (GC) is the fifth most common cancer worldwide, with nearly one million new diagnoses every year, and is the third leading cause of cancer-related deaths (1, 2). Most patients have the middle- or late-stage disease when diagnosed, and have very short survival and high mortality (3). Globally, GC is characterized remarkable by two major topographical subsites, cardia (CGC) and non-cardia (NCGC) (4). CGC and NCGC have different clinical biological characteristics in different geographical, ethnic and socioeconomic groups. Therefore, CGC and NCGC prognoses remain controversial. Some studies have reported that patients with CGC have a worse prognosis, while others have found no significant differences between the two subtypes (5, 6). It is of great importance to study the relationship between the pathogenesis and location of GC, as these factors can contribute to the development of new therapeutic strategies to improve patient prognosis.

Tumor-associated macrophages (TAMs) are macrophages that infiltrate tumor tissues and are among the most abundant immune cells in the tumor microenvironment (TME). Two main functional subtypes of macrophages have been described: M1 and M2. The function of M1-like macrophages include antigen presentation and tumor cell destruction, while M2-like macrophages promote extracellular matrix remodeling and angiogenesis and exhibit immunomodulatory characteristics (7). The anti-inflammatory characteristics of TAMs are controlled by tumor cells, and this is of great significance in treatment strategies for patients with GC, especially for combination therapies that target cancer cells and macrophages, which can have synergistic effects (8). Moreover, the efficacy of PD-L1/PD-1 antibodies in GC requires M1-like TAMs because they recruit more infiltrating CXCR3^+^CD8^+^ T cells by releasing CXCL9, 10, and 11 (9). M1-like TAMs not only improve the objective response rates of GC but also increase the application of PD-L1/PD-1 antibodies. This suggests that TAMs play a key role in GC development and provide a good target for anticancer therapy (9, 10). Previous studies have shown that high levels of TAM infiltration relate to the aggressive characteristics of GC and are independent poor prognostic factors for patients with GC (11, 12). Nevertheless, no prognostic difference was observed between CD68 density and overall survival (OS) in another study (13) and the M2-like macrophage signature has also been associated with improved survival (14). This could be because TAMs at different locations within the tumor differentially impact GC progression (15). Therefore, we speculated that the number and distribution of TAMs are key factors that affect the coevolution of cancer cells and TAMs.

In tumors, TAMs are usually stimulated by environmental factors to differentiate into M2-like macrophages. Due to the scavenging capabilities of M2 cells, the scavenger receptor CD163 has been proposed as a marker for M2-like macrophages. CD68 is considered the gold standard marker for human macrophages (16). Thus, in this study, we selected CD68 (pan-macrophage marker) and CD163 (M2-like macrophage) labeled TAM to explore the relationship between TAM infiltration at different locations and the prognosis of patient with GC.

*Helicobacter pylori* (*H. pylori*) is the most common chronic bacterial infection, affecting approximately 50% of the world’s population, and is a major risk factor for the development of GC (17). It was previously reported that *H. pylori* urease-activated mucosal macrophages can produce proinflammatory cytokines, which result in *H. pylori*-related mucosal inflammation (18). In mice with colitis-associated cancer, *H. pylori* infection was found to reduce TAM infiltration, especially the infiltration of M2-like TAMs (19). Che et al. also demonstrated that *H. pylori* infection-induced upregulation of activated mesenchymal–epithelial transition factor in exosomes influences the tumor-promoting effect of TAMs (20). These findings suggest a connection between *H. pylori* and TAMs.

In this study, we explored the relationship between TAM infiltration in different locations and the prognosis of patients with GC, and also whether TAM infiltration associates with *H. pylori* infection. We found that the distribution of TAMs has distinct characteristics in CGCs and NCGCs. In CGC, CD68^+^ and CD163^+^ macrophages were distributed at the invasive margin (MI) in most samples, while they mainly existed at the core (CT) in the NCGC group. The relationship between TAMs in different locations and the prognosis of patients with GC is conflicting. We thus utilized the difference to distinguish GC into central and marginal GC, to determine the relationship more accurately between macrophages and the prognosis of patients with GC. Moreover, a positive *H. pylori* status in central GC was significantly associated with better prognosis and TAM infiltrations. Taken together, these results suggest that TAMs and *H. pylori* are independent prognostic and predictive biomarkers for GC, and this finding might shed light on a new potential target for immunotherapeutic approaches for treating GC.

## Materials and Methods

### Study Subjects

This study retrospectively evaluated 200 stage III GC samples (100 cases of CGC and 100 cases of NCGC) from patients who underwent resection at the Tianjin Medical University Cancer Institute and Hospital between January 2012 and December 2014. None of the patients had received chemotherapy or radiotherapy before surgery. Patients with infectious diseases, autoimmune diseases or multiple primary cancers were excluded. All procedures were approved by the Ethics Committee of the Tianjin Medical University Cancer Institute and Hospital, and informed consent was obtained from all the subjects.

### Immunohistochemistry Staining

Paraffin-embedded slides were dewaxed in xylene and ethanol and steamed in a microwave oven with pH 9.0 ethylenediamine tetraacetic acid or pH 6.0 sodium citrate buffer to retrieve antigen epitopes. Once the samples had cooled, endogenous peroxidase activity was blocked using 3% hydrogen peroxide and the samples were blocked using goat serum. Slides were then incubated at 4°C overnight with the following primary antibodies: anti-human CD68 antibody (dilution 1:400, Invitrogen, USA), anti-human CD163 antibody (dilution 1:200, Abcam, Cambridge, UK), and anti-*H. pylori* antibody (dilution 1:10, MXB Biotechnology, China). The next day, a secondary antibody was labeled with streptavidin–horseradish peroxidase and was applied to the sample, using a DAB staining kit (MXB Biotechnology).

### Immunohistochemistry Evaluation

The number and distribution of TAMs were evaluated to assess the role of TAMs in GC progression. For each tissue section, eight fields of view (four MI and four CT) were selected randomly for histological evaluation by two pathologists who were blinded to the clinical characteristics of the patients. First, CD68 and CD163 immunohistochemical staining was calculated from the number of positive cells, and the average number was recorded as the number of TAMs. Second, the geographic distributions of TAMs were evaluated to uncover the role of TAMs in different locations. Next, the *H. pylori* status (negative or positive) was evaluated on the antral mucosa and corpora, which were stripped along the lesser curvature side. The degree of *H. pylori* infection was calculated by counting the number of bacteria (*H. pylori*) in each oil immersion field. The two pathologists randomly selected four fields of view to obtain the average value that was used to assess the relationship with the TAMs.

### Statistical Analysis

IBM SPSS Statistics Software, version 20, was used for all statistical analyses. All p-values were two-sided, and the statistical significance cutoff was p ≤0.05. The χ^2^ test was used to assess the relationship between clinicopathologic features of patients and CD68^+^ macrophages, CD163^+^ macrophages, and *H. pylori* infection status. Kaplan–Meier survival analysis was performed using ‘low’ or ‘high’ classifications according to the median number of CD68^+^ and CD163^+^ macrophages, and *H. pylori* status was based on the presence or absence of infection. Cox regression proportional hazard models were used to quantify hazard ratios for death from GC in both univariate and multivariate analyses. The models were adjusted for macrophages, *H. pylori*, age, sex, body mass index, carcinoembryonic antigen, tumor location, tumor size, and lymph node metastasis. The correlation between the number of TAMs and the degree of *H. pylori* infection was estimated using Spearman’s correlation analysis.

## Results

### Distinct Distribution of Macrophages in CGC and NCGC

**Figure 1:**
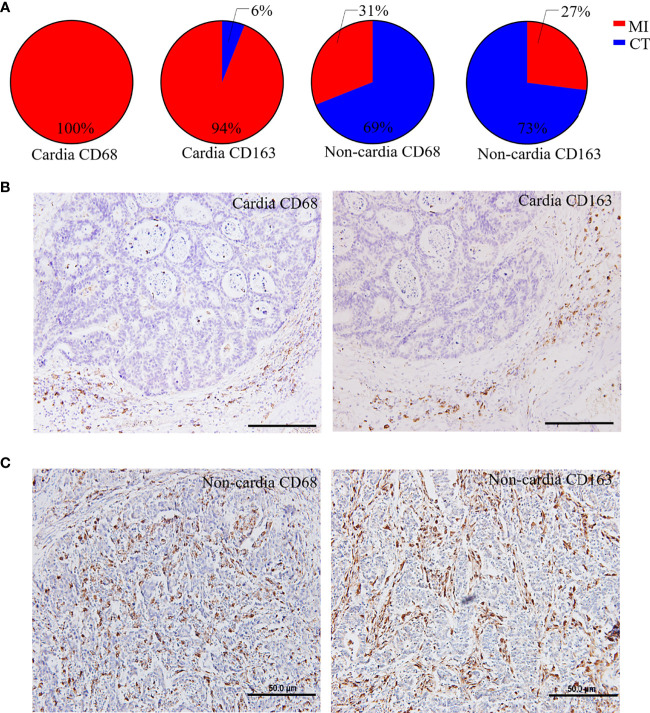
Distinct distribution of macrophages in CGC and NCGC. **(A)** The proportion of the distribution type of CD68^+^ or CD163^+^ macrophages in cardia and non-cardia tumor tissue samples, respectively. Representative picture of immunohistochemical staining of CD68^+^ or CD163^+^ macrophages in cardia **(B)** and non-cardia **(C)** gastric cancer tissues. CT, core of primary tumors; MI, invasive margin of primary tumors.

A total of 200 patients with GC participated in this study, and their clinicopathological characteristics are shown in **Table 1**. The median OS and disease-free survival (DFS) of the patients were 47.5 and 33 months, respectively and the average age was 59.8 years (range, 30–83 years). TAMs were widely distributed in the tumor tissues of patients with GC, and it was clearly observed that their distribution had distinct characteristics in CGC and NCGC cohorts, especially with respect to differences between CT and MI regions. In CGC, the number of CD68^+^ and CD163^+^ macrophages at the MI in most samples was significantly higher than that in the CT (**Figures 1A, B**). In contrast, their number was markedly higher at the CT of the NCGC cohort than that at the MI (**Figures 1A, C**). In other words, CD68^+^ and CD163^+^ macrophages in CGC were primarily distributed at the MI, while they mainly existed at the CT in the NCGC. These findings suggest CD68^+^ and CD163^+^ macrophages distributions may play an important role in determining the prognosis of patients with GC subtypes.

**Table 1:** Correlation analysis between macrophages, Hp infection and clinicopathologic features of the patients with gastric cancer.

Variables	All cases	CD68^+^ macrophages (CT)		P value	CD68^+^ macrophages (MI)		P value	CD163^+^ macrophages (CT)		P value	CD163^+^ macrophages (MI)		P value	Hp		P value
		Low	High		Low	High		Low	High		Low	High		Low	High	
Age																
<50 y	38		23 (60.5%)	0.108	17 (44.7%)	21 (55.3%)	0.474	16 (42.1%)	22 (57.9%)	0.151	15 (39.5%)	23 (60.5%)	0.207	9 (23.7%)	29 (76.3%)	1
≥50 y	162	88 (54.3%)	74 (45.7%)		84 (51.9%)	78 (48.1%)		90 (55.6%)	72 (44.4%)		85 (52.5%)	77 (47.5%)		40 (24.7%)	122 (75.3%)	
Sex																
Female	51	27 (52.9%)	24 (47.1%)	0.872	25 (49.0%)	26 (51.0%)	0.872	24 (47.1%)	27 (52.9%)	0.335	28 (54.9%)	23 (45.1%)	0.517	13 (25.5%)	38 (74.5%)	0.852
Male	149	76 (51.0%)	73 (49.0%)		76 (51.0%)	73 (49.0%)		82 (55.0%)	67 (45.0%)		72 (48.3%)	77 (51.7%)		36 (24.2%)	113 (75.8%)	
BMI (kg/m2)																
Low	114	56 (49.1%)	58 (50.9%)	0.477	57 (50.0%)	57 (50.0%)	0.887	62 (54.4%)	52 (45.6%)	0.67	53 (46.5%)	61 (53.5%)	0.317	25 (21.9%)	89 (78.1%)	0.407
High	86	47 (54.7%)	39 (45.3%)		44 (51.2%)	42 (48.8%)		44 (51.2%)	42 (48.8%)		47 (54.7%)	39 (45.3%)		24 (27.9%)	62 (72.1%)	
CEA																
<5 µg/L	56	28 (50.0%)	28 (50.0%)	0.875	25 (44.6%)	31 (55.4%)	0.346	28 (50.0%)	28 (50.0%)	0.638	26 (46.4%)	30 (53.6%)	0.637	14 (25.0%)	42 (75.0%)	1
≥5 µg/L	144	75 (52.1%)	69 (47.9%)		76 (52.8%)	68 (47.2%)		78 (54.2%)	66 (45.8%)		74 (51.4%)	70 (48.6%)		35 (24.3%)	109 (75.7%)	
Tumor location																
Cardia	100	52 (52.0%)	48 (48.0%)	1	51 (51.0%)	49 (49.0%)	1	55 (55.0%)	45 (45.0%)	0.671	50 (50.0%)	50 (50.0%)	1	34 (34.0%)	66 (66.0%)	0.003
Non-cardia	100	51 (51.0%)	49 (49.0%)		50 (50.0%)	50 (50.0%)		51 (51.0%)	49 (49.0%)					15 (15.0%)	85 (85.0%)	
Tumor size											52 (54.7%)	43 (45.3%)	0.257			
<4 cm	95	38 (40.0%)	57 (60.0%)	0.003	58 (61.1%)	37 (38.9%)	0.005	46 (48.4%)	49 (51.6%)	0.257	48 (45.7%)	57 (54.3%)		21 (22.1%)	74 (77.9%)	0.512
≥4 cm	105	65 (61.9%)	40 (38.1%)		43 (41.0%)	62 (59.0%)		60 (57.1%)	45 (42.9%)					28 (26.7%)	77 (73.3%)	
LN metastasis											4 (66.7%)	2 (33.3%)	0.683			
No	6	2 (33.3%)	4 (66.7%)	0.434	3 (50.0%)	3 (50.0%)	1	5 (83.3%)	1 (16.7%)	0.217	96 (49.5%)	98 (50.5%)		0 (0.0%)	6 (100.0%)	0.339
Yes	194	101 (52.1%)	93 (47.9%)		98 (50.5%)	96 (49.5%)		101 (52.1%)	93 (47.9%)					49 (25.3%)	145 (74.7%)	

### High Levels of CD68^+^ Macrophage Infiltration in the CT Associate With a Better Prognosis in CGC, Whereas Infiltration at the MI Indicates a Poor Prognosis in NCGC

**Figure 2:**
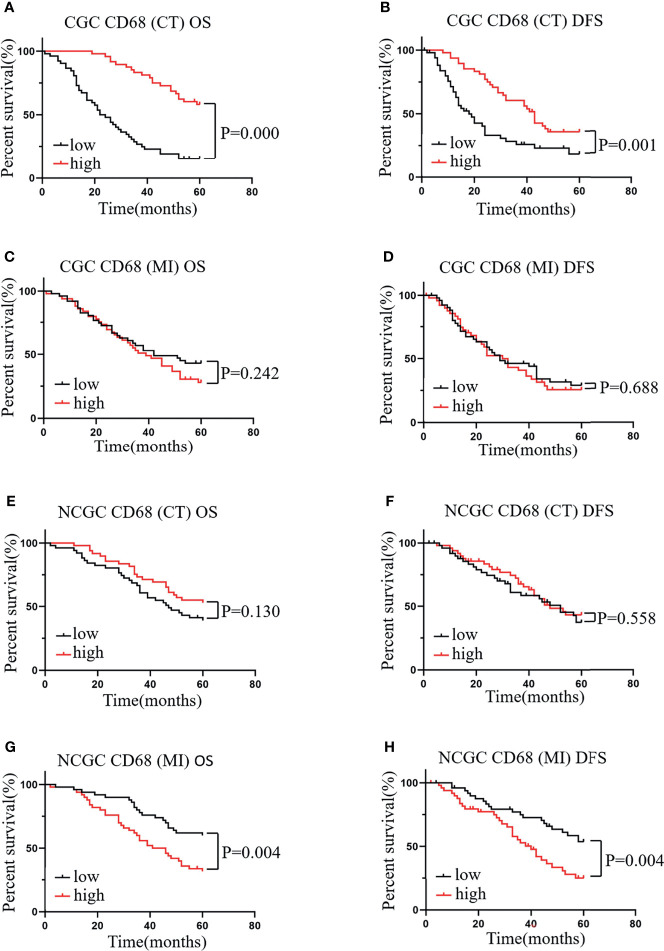
The relationship between CD68^+^ macrophages in different locations and prognosis and clinicopathological features of patients with CGC and NCGC. **(A, B)** A higher number of CD68^+^ macrophages at the CT was associated with better OS and DFS in CGC. **(C, D)**. A higher number of CD68^+^ macrophages at MI not correlated with OS or DFS in CGC. **(E, F)** A higher number of CD68^+^ macrophages at the CT was not correlated with OS or DFS in NCGC. **(G, H)** A higher number of CD68^+^ macrophages at the MI was closely correlated with poor OS and DFS in NCGC. CT, core of primary tumors; MI, invasive margin of primary tumors.

To determine the effects of the distribution of TAMs on GC, we first assessed the role of CD68^+^ macrophages on CT and MI in patients with CGC and NCGC. We found that higher CD68^+^ macrophage infiltration at the CT associated with better OS and DFS in CGCs (**Figures 2A, B**). However, CD68^+^ macrophage infiltration at the MI did not correlate with OS or DFS in CGCs (**Figures 2C, D**). Furthermore, in the NCGC cohort, a higher CD68^+^ macrophage infiltration at the CT did not correlate with OS or DFS (**Figures 2E, F**). Interestingly, higher CD68^+^ macrophage infiltration at the MI closely correlated with poor OS and DFS in NCGCs (**Figures 2G, H**).

Next, we analyzed the relationship between the number of CD68^+^ macrophages and clinicopathological features. As shown in **Table 1**, no association was observed between the number of CD68^+^ macrophages that infiltrated the CT and MI regions and the clinicopathological features of patients, including age, sex, body mass index, carcinoembryonic antigen, tumor location, and lymph node metastasis, with the exception of tumor size. These results indicate that the higher the level of CD68^+^ macrophage infiltration at the CT in CGC, the better the prognosis of the patients. Conversely, the higher the level of CD68^+^ macrophage infiltration at the MI in NCGC, the worse the prognosis of the patients.

### High Levels of CD163^+^ Macrophage Infiltration at the MI correlate With a Poor Prognosis of Patients With CGC and NCGC

**Figure 3:**
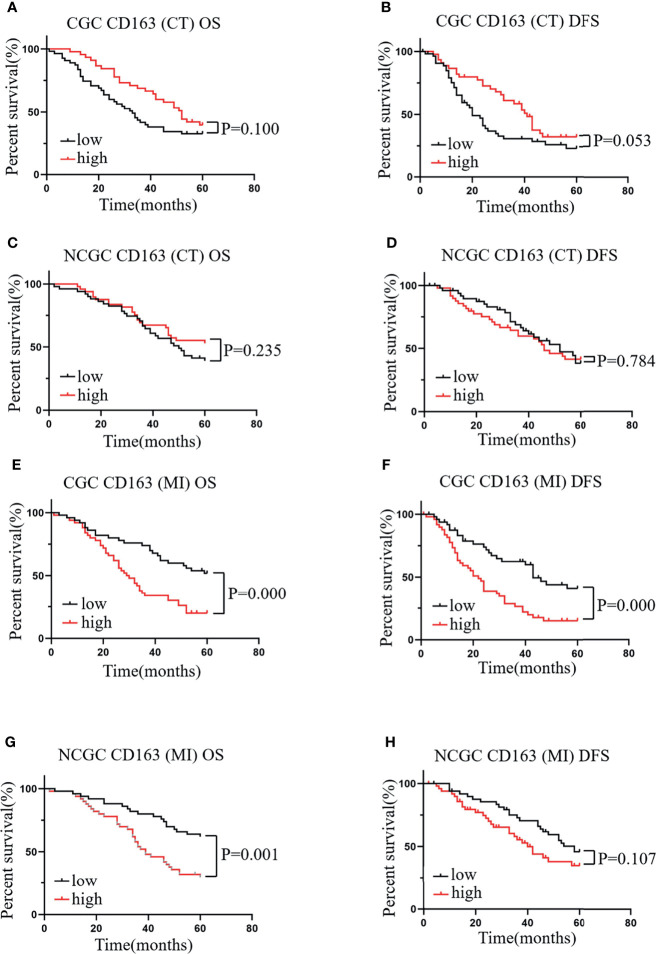
The relationship between CD163^+^ macrophages in different locations and prognosis and clinicopathological features of patients with CGC and NCGC. A higher number of CD163^+^ macrophages at the CT was not correlated with OS or DFS in CGC **(A, B)** or NCGC **(C, D)**. **(E, F)** A higher number of CD163^+^ macrophages at the MI was associated with poor OS and DFS in CGC. **(G, H)** A higher number of CD163^+^ macrophages at the MI was closely correlated with poor OS in NCGC, while DFS was not. CT, core of primary tumors; MI, invasive margin of primary tumors.

Similarly, we evaluated the relationship between CD163^+^ macrophages infiltration at the CT and MI regions and the prognosis of patients with CGC or NCGC. The results showed that higher CD163^+^ macrophage infiltration at the CT did not correlate with OS and DFS in CGC (**Figures 3A, B**) or NCGC (**Figures 3C, D**). This differed from the impact of CD163^+^ macrophage infiltration at the MI. A higher CD163^+^ macrophage infiltration at the MI in patients with CGC associated with poor OS and DFS (**Figures 3E, F**). Furthermore, a higher CD163^+^ macrophage infiltration at the MI also closely correlated with poor OS in NCGCs (**Figure 3G**), but not with DFS (**Figure 3H**). In addition, the number of CD163^+^ macrophages that infiltrated the CT and MI did not associate with the clinicopathological features of patients with GC (**Table 1**). In summary, a higher CD163^+^ macrophage infiltration at the MI in patients associated with a poor prognosis for CGC and NCGC.

### Patients With Central GC Have Better OS and DFS

**Figure 4:**
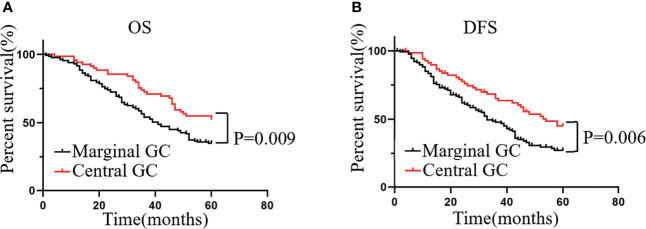
Patients with central GC have better OS and DFS. **(A, B)** Kaplan–Meier analysis graph showing that patients with marginal GC had poor OS and DFS.

Considering the large differences in the relationship between the infiltration of CD68^+^ macrophages at different locations and prognosis, we used these differences as the basis for a classification system to estimate the prognosis of patients more accurately with GC. If the number of CD68^+^ macrophages that infiltrated the MI was greater than that at the CT, the sample was defined as “marginal GC”; otherwise, it was defined as “central GC”. We found that, compared with the prognosis of patients with marginal GC, patients with central GC had a better OS (**Figure 4A**) and DFS (**Figure 4B**). That is, patients with more CD68^+^ macrophages infiltration at the CT had a better prognosis in the central GC cohort.

### Positive *H. pylori* Status in Central GC Significantly Associates With Better Prognosis and TAM Infiltration

**Figure 5:**
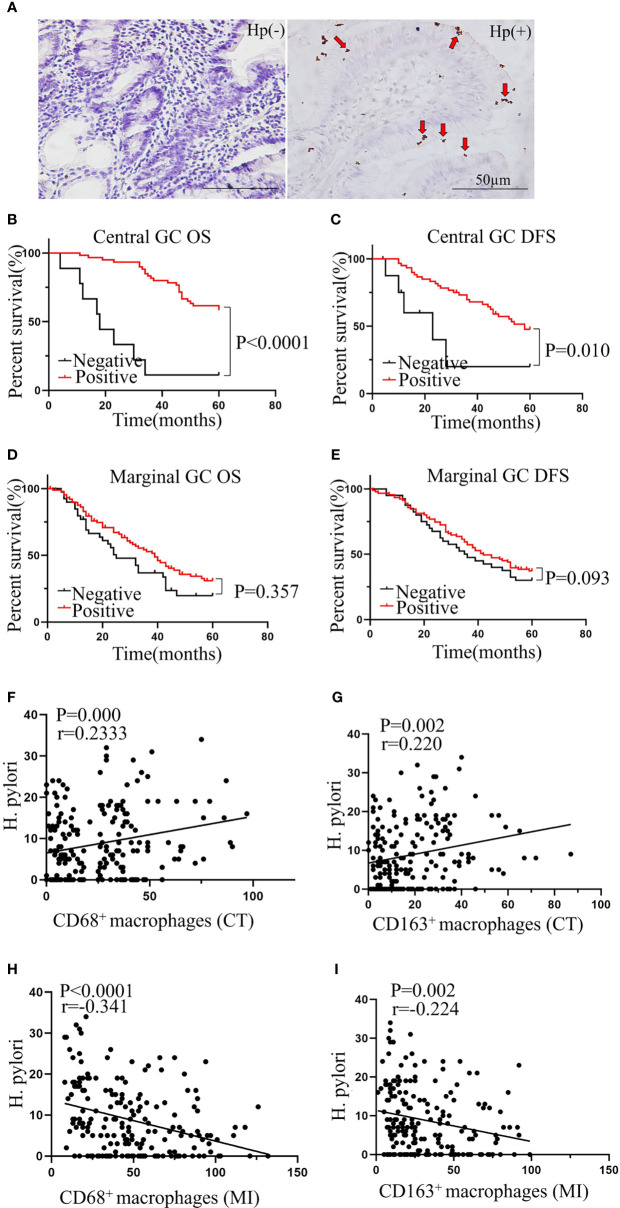
Positive *H. pylori* status in central GC was significantly associated with better prognosis and TAM infiltration. **(A)** Representative picture of immunohistochemical staining of *Helicobacter pylori*. **(B, C)** Positive *H. pylori* status was significantly associated with better OS and DFS in central GC and was not associated with better OS and DFS in marginal GC **(D, E)**. **(F–I)** The correlation between the TAMs in different locations and *H. pylori*. CT, core of primary tumors; MI, invasive margin of primary tumors.

*H. pylori* infection has been reported to play a key role in GC (21, 22) and has been found to relate to macrophage infiltration in a mouse model (19). Therefore, we hypothesized that the infiltration of TAMs in cancer may be related to *H. pylori* infection. We first evaluated the status of *H. pylori* infection in GC patients using immunochemistry (**Figure 5A**). We next analyzed the relationship between *H. pylori* infection status and the clinicopathological features of patients with GC. We found that *H. pylori* infection status related to tumor location, but not tumor size, age, sex, body mass index, carcinoembryonic antigen, or lymph node metastasis (**Table 1**). Further survival analysis showed that a positive *H. pylori* status in the central GC group was significantly associated with better OS and DFS (**Figures 5B, C**). However, there was no correlation with marginal GC (**Figures 5D, E**). Moreover, we detected a correlation between macrophages at different locations and the degree of *H. pylori* infection. As expected, the degree of *H. pylori* infection positively correlated with the infiltration of CD68^+^ and CD163^+^ macrophages in the CT region (**Figures 5F, G**), whereas *H. pylori* infection negatively correlated with the infiltration of CD68^+^ and CD163^+^ macrophages at the MI (**Figures 5H, I**).

Finally, we constructed Cox proportional hazards models between clinical outcomes and survival in patients with GC. The univariable analysis revealed that the level of macrophages in different locations and tumor size related to the prognosis of patients with GC (**Table 2**). Further analysis of multivariable Cox regression showed that CD163^+^ macrophage infiltration at the MI and tumor size were independent high-risk factors, whereas CD68^+^ macrophage infiltration at the CT and *H. pylori* positivity had a protective effect (**Table 2**). Hence, the above data indicate that TAMs in different locations and *H. pylori* infection were independent prognostic markers, and with an obvious correlation between them.

**Table 2:** Cox proportional hazards models between clinical and survival in patients with gastric cancer.

Variables	Univariable		Multivariable	
	HR(95% CI)	P value	HR(95% CI)	P value
CD68^+^ macrophages (CT)	0.399 (0.274—0.581)	0.000	0.364 (0.232—0.570)	0.000
CD68^+^ macrophages (MI)	1.691 (1.171—2.441)	0.005		
CD163^+^ macrophages (CT)	0.690 (0.479—0.994)	0.046		
CD163^+^ macrophages (MI)	2.459 (1.684—3.589)	0.000	2.644 (1.676—4.174)	0.000
Hp (Positive vs. Negative)	0.541 (0.365—0.801)	0.002	0.589 (0.387—0.896)	0.013
Age (≥50y vs. <50y)	1.206 (0.752—1.932)	0.437		
Sex (Male vs. Female)	1.058 (0.699—1.602)	0.789		
Body mass index (High vs. Low)	0.952 (0.661—1.372)	0.792		
CEA (≥5 µg/L vs. <5 µg/L)	1.047 (0.698—1.572)	0.823		
Tumor location (Non-cardia vs. Cardia)	1.410 (0.981—2.026)	0.063		
Tumor size (≥4 cm vs. <4 cm)	2.361 (1.615—3.451)	0.000	2.047 (1.383—3.031)	0.000
LN metastasis (Yes vs. No)	5.260 (0.735—37.670)	0.098		

## Discussion

GC, currently ranked fourth in global cancer-related mortality worldwide, is often diagnosed when it reaches an advanced stage after distant metastasis (23). The importance of TAMs in the development of GC is gaining an increasing interest (24). However, the precise role of TAMs in GC remains unknown and even somewhat contradictory. Here, we revealed a clear effect of different TAM infiltration phenotypes at the CT and MI on GC with topographical subsites. We found a discrepancy in the relationship between TAMs at different locations and the prognosis of patients with GC. Most importantly, positive *H. pylori* infection in central GC was significantly associated with better prognosis and TAM infiltrations. These findings contribute to a more complete understanding of the correlation between TAMs and the prognosis of patients with GC and have important implications for clarifying their potential role as therapeutic targets.

The densities and prognostic effects of tumor-infiltrating lymphocytes differ in relation to tumor locations within the stomach (25). There are also contrasting prognostic effects of the Foxp3/CD4 ratio in CGC and NCGC (25), which suggests that location may be an important factor for tumor progression. It is possible that neglecting the location during risk assessment could account for the differential findings in previous studies (26, 27). Indeed, accumulating evidence has demonstrated distinct molecular and pathophysiological mechanisms of carcinogenesis in CGC and NCGC (28, 29). Different staging systems have also been used to assess CGC and NCGC (30). Moreover, significant advances have been made in studies of the impact of TAMs on clinical outcomes, and the clinical significance of TAMs can be affected by their number, phenotypes, and distributions at each pathological stage. Therefore, we sought to determine the impact of different macrophages infiltration phenotypes at the CT or MI on the prognosis of patients with CGC or NCGC.

Our findings revealed the distinct TAMs distribution characteristics in CGCs and NCGCs. In most samples of CGC, CD68^+^ and CD163^+^ macrophages were distributed at the MI, whereas they mainly existed at the CT in the NCGC samples. The Kaplan–Meier analysis showed that a high number of CD68^+^ macrophages at the CT in CGCs was associated with a better prognosis. Conversely, a high number of CD68^+^ macrophages at the MI in NCGCs was associated with a poor prognosis. These data indicate the differential effects of CD68^+^ macrophage infiltration at different sites on the prognosis of GC. This may also partly explain why certain prior studies have shown that a high density of CD68^+^ TAMs predicts a poor prognosis in GC (31), while other studies have demonstrated no prognostic difference of CD68 density on OS (13). TAMs at different sites in GC tissue might represent a distinct significance and prognostic value (32).

In addition, the specific localization of TAMs is affected by the environment at different areas in the tumor tissue, and TAM functions have been inconsistent, such as in the presence of hypoxia (33). In breast cancer, tumor nest-associated macrophages have been found to promote angiogenesis to a greater extent than macrophages in the tumor stroma (34). Similarly, our results found that tumor nest-associated CD68^+^ macrophages and tumor stroma-associated CD68^+^ macrophages have different prognostic values for patients with GC, which suggests that macrophages infiltration at the CT or MI have different TME roles. For another, CD68^+^ macrophages at the CT or MI whether have more complex phenotypes. The M1/M2 paradigm represents two extreme TAM activation states, which may neglect that the flexible, rather than static, adaptation driven by environmental signals in the TME. To distinguish the unique role of TAMs under various conditions, it is urgent to redefine TAM subsets and their function in TMEs. A better understanding of how TAM subsets are affected by conditions in specific regions will certainly benefit the related treatments.

*H. pylori* infection greatly promotes the carcinogenic effects of GC. Interestingly, many studies have reported that a positive *H. pylori* infection status predicts the survival of patients with GC, with a favorable effect. A prospective study showed that patients with GC with *H. pylori* infections had better OS and DFS after radical resection (35). A meta-analysis (36) of 2,454 patients also showed that *H. pylori* infection is an independent protective factor for GC progression, and this protective effect applies to different ethnicities (36). These findings are also consistent with our results, which show that positive *H. pylori* status in central GC was significantly associated with better prognosis. However, further experiments are required to verify this mechanism (37).

The suppressive effect of *H. pylori* on GC progression may be due to the induction of an improved immune response against the tumor (35, 38). It has been suggested that *H. pylori* components simulate surface molecules or specific receptors on gastric epithelial cells, and autoantibodies can induce a cross-reaction against GC cells (39). Another possible explanation is that the true prognostic significance of *H. pylori* status may be suspected, as a negative *H. pylori* infection status may only represent a more advanced tumor status. Hobsley et al. (39) proposed that with GC progression, most or all parietal cells become destroyed in advanced GC, which results in the stomach becoming alkaline and negative for *H. pylori* infection, while *H. pylori* is positive in patients with early and milder GC. Nevertheless, in our study, we selected patients with stage III advanced GC, and the *H. pylori* infection positivity rate was 67.6%, which is relatively high. Our results also showed that *H. pylori* infection is closely related to macrophages infiltration. Considering previous reports and our current research, we are more inclined to hypothesize that *H. pylori* status has strong prognostic significance. Since *H. pylori* in the stomach can continuously release bacterial components into the gastrointestinal tract, *H. pylori* components in the cavity may interact with immune cells (40). Indeed, a number of studies have demonstrated the effect of *H. pylori* infection on macrophages polarization through *in vivo* and *in vitro* experiments. *H. pylori* not only prevents chronic colitis by promoting M2 polarization (41), but also promotes M1 polarization of human and mouse gastric macrophages, resulting in the occurrence of *H. pylori*-related atrophic gastritis (42). In addition, Lu et al. (43) confirmed that a low *H. pylori* multiplicity of infection (MOI) of promotes the M1 and M2 phenotypes, while a high MOI suppresses the M2 phenotype. Intriguingly, our current research focuses on a novel perspective that the degree of *H. pylori* infection relates to the CD68^+^ and CD163^+^ macrophage infiltration at different locations in GC tissue. However, the mechanism underlying this effect remains to be elucidated. Towards better understanding the underlying mechanism, and verifying the influence of *H. pylori* infection on the number, location, and polarization of tumor tissue infiltrating macrophages, an animal model of GC has been established. Furthermore, *in vitro* experiments are underway to determine whether *H. pylori* is itself a key factor. We hope that our future research will address these outstanding questions.

In summary, our research differs from prior studies in that it focuses on the role of *H. pylori* and TAM infiltration on GC according to the topographic locations of tumors within the stomach. Our results suggest a new classification method based on CD68^+^ macrophage infiltration at the CT to evaluate the prognosis of patients more accurately with GC. We found that CD68^+^ macrophage infiltration in the CT and a positive *H. pylori* status were independent protective factors in central GC. These findings indicate that tumor location along with the location of infiltrating cells within the stomach should be considered when evaluating individualized patient prognosis. Furthermore, elucidating the detailed connection between *H. pylori* and TAMs will facilitate the development of new therapeutic strategies.

## Data Availability Statement

The raw data supporting the conclusions of this article will be made available by the authors, without undue reservation.

## Ethics Statement

The studies involving human participants were reviewed and approved by the Ethics Committee of Tianjin Medical University Cancer Institute and Hospital. The patients/participants provided their written informed consent to participate in this study.

## Author Contributions

QS and XR conceived and designed the experiments. ML, LZ, QZ, and YW performed the experiments. XR provided some suggestions. Data analysis was performed by YW and ML. Writing, reviewing, and manuscript editing was done by ML, QS, and XR. All authors contributed to the article and approved the submitted version.

## Funding

This work was supported by grants from the National Key R&D Program of China (2018YFC1313400), the National Natural Science Foundation of China (81974416, 81872166, U20A20375 and 81802873), the Tianjin Natural Science Foundation (19JCYBJC27600 and 18JCQNJC81300), and the Scientific Research Program of Tianjin Education Commission (2019KJ185).

## Conflict of Interest

The authors declare that the research was conducted in the absence of any commercial or financial relationships that could be construed as a potential conflict of interest.

## Publisher’s Note

All claims expressed in this article are solely those of the authors and do not necessarily represent those of their affiliated organizations, or those of the publisher, the editors and the reviewers. Any product that may be evaluated in this article, or claim that may be made by its manufacturer, is not guaranteed or endorsed by the publisher.
